# Randomized controlled trial of vitamin D supplementation in older people to optimize bone health

**DOI:** 10.1093/ajcn/nqy280

**Published:** 2019-01-08

**Authors:** Terry J Aspray, Thomas Chadwick, Roger M Francis, Elaine McColl, Elaine Stamp, Ann Prentice, Alexander von Wilamowitz-Moellendorff, Inez Schoenmakers

**Affiliations:** 1 NIHR Newcastle Biomedical Research Center, Campus for Aging and Vitality, Newcastle upon Tyne, United Kingdom; 2 Institute for Cellular Medicine, Newcastle University, Newcastle upon Tyne, United Kingdom; 3 Institute of Health and Society, Newcastle University, Newcastle upon Tyne, United Kingdom; 4 MRC Elsie Widdowson Laboratory, Cambridge, United Kingdom; 5 Norwich Medical School, Norwich, United Kingdom

**Keywords:** vitamin D, older people, randomized controlled trial, bone mineral density, dual-energy X-ray absorptiometry

## Abstract

**Background:**

Vitamin D insufficiency is common in older people and may lead to increased bone resorption, bone loss, and increased falls and fractures. However, clinical trials assessing the effect of vitamin D supplementation on bone mineral density (BMD) have yielded conflicting results.

**Objectives:**

This study examined the effect of vitamin D supplementation on BMD at the hip, using dual-energy X-ray absorptiometry.

**Methods:**

A total of 379 adults aged ≥70 y (48% women; mean age: 75 y) from the northeast of England were randomly allocated to 1 of 3 doses of vitamin D_3_ [12,000 international units (IU), 24,000 IU, or 48,000 IU] given once a month. The primary outcome was change in BMD (ΔBMD) at the hip. Secondary endpoints comprised the dose effects on femoral neck BMD, falls, circulating calciotropic hormones, bone turnover markers, and adverse events.

**Results:**

The mean ± SD baseline plasma 25-hydroxyvitamin D [25(OH)D] concentration was 40.0 ± 20.1 nmol/L, which increased after 12 mo to a mean 25(OH)D of 55.9, 64.6, or 79.0 nmol/L for participants receiving a monthly dose of 12,000, 24,000, or 48,000 IU, respectively (*P* < 0.01 for difference). There was no between-group difference in ΔBMD. However, parathyroid hormone concentrations decreased in all 3 groups, with a significantly greater decrease in the 48,000-IU group compared with the 12,000-IU group (*P* < 0.01). There were no differences in any adverse events between groups, with 3 cases of hypercalcemia, none of nephrolithiasis, and 249 falls observed.

**Conclusions:**

There was no difference in change in BMD over 12 mo between the 3 doses of vitamin D, suggesting no effect of the intervention or a similar attenuation of the anticipated decrease in BMD over 12 mo. The treatment was safe and effective in increasing plasma 25(OH)D concentrations, with no dose-related adverse events. This trial was registered at the EU Clinical Trials Register (EudraCT 2011-004890-10) and the ISRCTN Registry (ISRCTN35648481).

## Introduction

Vitamin D insufficiency is common in older people and may lead to increased bone resorption, bone loss, impairment of muscle function, and an increased risk of falls and fractures. The results of clinical trials assessing the effect of vitamin D supplementation on bone mineral density (BMD), bone loss, falls, and fractures have yielded conflicting results, and, although a recent meta-analysis of clinical trials reported a possible relation between vitamin D supplementation and higher BMD at the neck of femur, it suggested that supplementation with vitamin D has a benefit for bone health only in those at risk of vitamin D deficiency (1).

Internationally, guidelines differ in their recommendations for vitamin D status for musculoskeletal health, as reflected by circulating 25-hydroxyvitamin D [25(OH)D] concentrations: in the United Kingdom, the Scientific Advisory Committee on Nutrition (SACN) recommends a concentration of ≥25 nmol/L (2). In North America, the Institute of Medicine (IOM) recommends a concentration of 50 nmol/L (3), whereas the Endocrine Society clinical guideline recommends a target concentration of 75 nmol/L for the maintenance of bone health and other nonskeletal benefit (4). The UK dietary Reference Nutrient Intake is 10 μg [400 international units (IU)]/d (2), whereas in North America, the Recommended Dietary Allowance (RDA) for those aged >70 y is 20 μg (800 IU)/d (3). However, in this age group in the United Kingdom, the mean daily vitamin D intake from dietary sources (including nutritional supplements) is ∼5.2 μg (208 IU)/d (2), and medical prescription of vitamin D supplements is relatively uncommon, even among those at highest risk (5, 6).

Decreased dose frequency has been identified as a factor associated with better adherence to pharmacological therapy (7), and because plasma 25(OH)D has a half-life estimated in terms of weeks rather than hours (8–10), daily dosing may not be required to maintain an adequate vitamin D status. However, decreasing dose frequency may have unanticipated effects. For example, one clinical trial evaluating an annual oral dose of 12,500 µg (500,000 IU) vitamin D found that there was an increase in falls and fractures (11).

Overall, previous study findings are conflicting, which may reflect variations in study design, the characteristics of participants (such as age, frailty, and baseline vitamin D status), and the nature of intervention, including vitamin D dose, its route, the frequency of administration, and the form of vitamin D (whether vitamin D_2_ or vitamin D_3_). The aim of this study was to measure the effect of vitamin D supplementation on the change in BMD at the hip in community-dwelling older people. We also investigated the effects of supplementation dose on a number of predefined secondary endpoints including change in plasma 25(OH)D [total and calculated free 25(OH)D], parathyroid hormone (PTH), and biochemical markers of bone turnover, as well as the frequency of falls and adverse events.

## Methods

The Vitamin D Supplementation in Older People (VDOP) Trial (ISRCTN35648481) was a single-center, parallel-group, participant-randomized, double-blind interventional trial testing the effects on hip BMD of 3 doses—300, 600, and 1200 µg (12,000, 24,000, and 48,000 IU)—of oral vitamin D_3_ given each month to men and women aged ≥70 y for 1 y as described earlier (12), with the first dose given between November 2012 and May 2013. The study was funded by Arthritis Research UK (D19544). Potential participants were identified from electronic medical records from 25 general practitioner practices in the northeast of England. Participants were invited to take part after ensuring they did not meet the following exclusion criteria:

- treatment with anti­resorptive or anabolic treatment for osteoporosis in the previous 3 y;
- current consumption of supplementary vitamin D at a dose >10 μg (400 IU)/d or calcium at a dose >500 mg/d;
- experience of a fragility fracture in the previous 6 mo;
- a history of renal stones, previous bilateral hip replacements, or primary hyperparathyroidism;
- past or present history of hypercalcemia (albumin ­adjusted plasma calcium >2.60 mmol/L), hypocalcemia (albumin­ adjusted plasma calcium <2.15 mmol/L), or an estimated glomerular filtration rate <30 mL · min^−1^ · 1.73 m^−2^.

Participants already taking elemental calcium supplements or vitamin D supplements in equivalent daily doses of ≤500 mg and 10 µg (400 IU), respectively, were allowed to continue their supplement. All participants provided written informed consent. A favorable opinion was obtained from the Tyne and Wear South Research Ethics Committee (REC, 12/NE/0050), with Research and Development approval from the sponsor, Newcastle upon Tyne Hospitals NHS Foundation Trust.

A total of 379 participants were recruited between November 2012 and May 2013. The first participant's first visit was on 8 November, 2012, and the last participant's last visit was on 6 June, 2014.

### Intervention

Participants were randomly assigned in a ratio of 1:1:1 to 1 of 3 doses of vitamin D_3_ taken orally each month for 1 y. A computer-generated allocation list of random permuted blocks was used to ensure concealment of allocation to subjects and investigators. Emergency code-break envelopes were retained at the trials pharmacy, but no code breaking was required during the study. The medications were identical in appearance so that neither participant nor investigator was aware of the dose given. The lowest dose (12,000 IU/mo) corresponded to an approximate daily dose of 10 µg (400 IU) and was used as a reference dose, equating to the Reference Nutrient Intake as defined by the SACN. The second dose (24,000 IU/mo) was equivalent to the North American Institute of Medicine's (IOM's) RDA of 800 IU/d for this age group (3, 13). The highest dose (48,000 IU/mo) is twice the IOM RDA and well below the Tolerable Upper Intake Level defined by the IOM of 4000 IU/d.

### Measurements

BMD at the total hip and neck of femur was measured using dual-energy X-ray absorptiometry (Lunar iDXA; GE Healthcare) at baseline and at 12 mo. The scanner was calibrated daily before participant scanning. Daily phantom measurements were performed, and precision on repeated measurements was 0.21%, with no perceptible drift for the duration of the study.

Height and weight were measured, and body composition was determined by bioelectrical impedance using a Tanita analyzer (Tanita Corp.) (14). Fracture risk and self-reported falls history were obtained at baseline, adapted from standard clinical care questions and the FRAX tool (15).

Participants kept a prospective falls diary with monthly prompts to record falls made by telephone, at which time they were also asked about adverse events (AEs), food supplements, and medication. Participants visited the clinical research facility every 3 mo, after an overnight fast, when blood and timed urine samples were collected and further information on AEs and questionnaires on sunshine exposure, diet, and quality of life were self-completed. Completed diaries and questionnaires were discussed with participants at each visit. For details on the study schedule, please refer to our previous publication (16).

### Biochemical analyses

Blood- and urine-sample collection and processing protocols, biochemical measurement, methods, and quality control of assay performance were strictly standardized, as previously described (12), except for those given below. The 25-hydroxyvitamin D_2_ [25(OH)D_2_] and 25-hydroxyvitamin D_3_ [25(OH)D_3_] concentrations were determined by liquid chromatography–tandem mass spectrometry (17). Total 25(OH)D concentration was determined by summing 25(OH)D_2_ and 25(OH)D_3_ concentrations. Assay performance was monitored using Chromsystems and in-house controls and is traceable to National Institute of Standards and Technology standards. The interassay variation was <10% for 25(OH)D_2_ and <7% for 25(OH)D_3_, and the limit of quantification was 6 nmol/L. External quality assessment was obtained through participation in Vitamin D External Quality Assessment Scheme (DEQAS; www.deqas.org).

Vitamin D binding protein (DBP) was measured by polyclonal antibody ELISA (Immunodiagnostik AG; Oxford Biosystems). All samples were measured in duplicate, with the exception of PTH, and the analysis was repeated if the CV was >10%. Assay performance was measured using kit and in-house controls, and performance was within acceptable limits. Free concentrations of 25(OH)D were calculated using published mathematical models (18) that included concentrations of total 25(OH)D, DBP, and albumin.

### Statistical analyses

The primary outcome for the study was the change in BMD at the hip in response to 12,000, 24,000, or 48,000 IU vitamin D_3_/mo for 1 y, using 12,000 IU as the reference dose, with and without adjustment for covariates selected from the following predetermined potentially confounding variables: presupplementation plasma 25(OH)D, PTH, weight, height, lean body mass, estimated glomerular filtration rate, 10-y fracture risk [FRAX (15)], gender, age, and baseline value of BMD. During the ANCOVA modeling process, covariates not reaching a threshold significance of 0.05 were removed, except for age, gender, weight, and height. The distribution of age was skewed and was included as a categorical variable based on age at recruitment grouped by quartile. Other outcomes were analyzed using this model with consideration of their baseline value rather than that of BMD.

To compare the absolute change in BMD at the hip, giving a 2-sided significance level of 0.05 and a power of 80%, 125 participants/arm were required. This resulted in a planned sample size of 375 participants to allow for 20% attrition by 12 mo. The estimate was based on the variance in change in BMD over 12 mo from a previous study (19) and enabling detection of a 0.006-g/cm^2^ difference (equivalent to a standardized effect size of 0.4) between 2 arms.

Details of the statistical analysis were predetermined (12) and constantly reviewed, with revision to evaluate the effects of circulating free 25(OH)D as well as total 25(OH)D concentrations to address recent development in the scientific field before unblinding of the data (20). A further post hoc analysis examined whether participants’ initial circulating 25(OH)D concentration being above or below 25 nmol/L as a categorical variable influenced the effect of the supplement and any interaction of this with trial arm. The statistical analysis plan (SAP 1.4) is available on request and incorporates a summary of predefined secondary outcomes, including total femur and femoral neck BMD, serum total 25(OH)D, plasma PTH, bone turnover markers, falls, and post hoc analyses outlined in this article, with AE reporting and the statistical analytical approach adopted. Although no formal correction for multiple testing has been applied during the analysis, consideration of Bonferroni correction was made in the Results and Discussion.

### Participant involvement in the study

At the design phase, the research team discussed the intentions of the study, the research questions and measurements, and burden to participants with a representative group of older adults recruited through the Bone Clinic, Freeman Hospital. There were also 2 lay members on the trial steering group. At the end of the study, a newsletter was sent to all participants outlining the results of the study, and the research team answered calls from interested participants. There was no formal assessment of the burden of the intervention on health care workload during the study, but we did collect data on functional outcomes and quality of life (not included in the primary analysis and yet to be analyzed).

## Results

**FIGURE 1:**
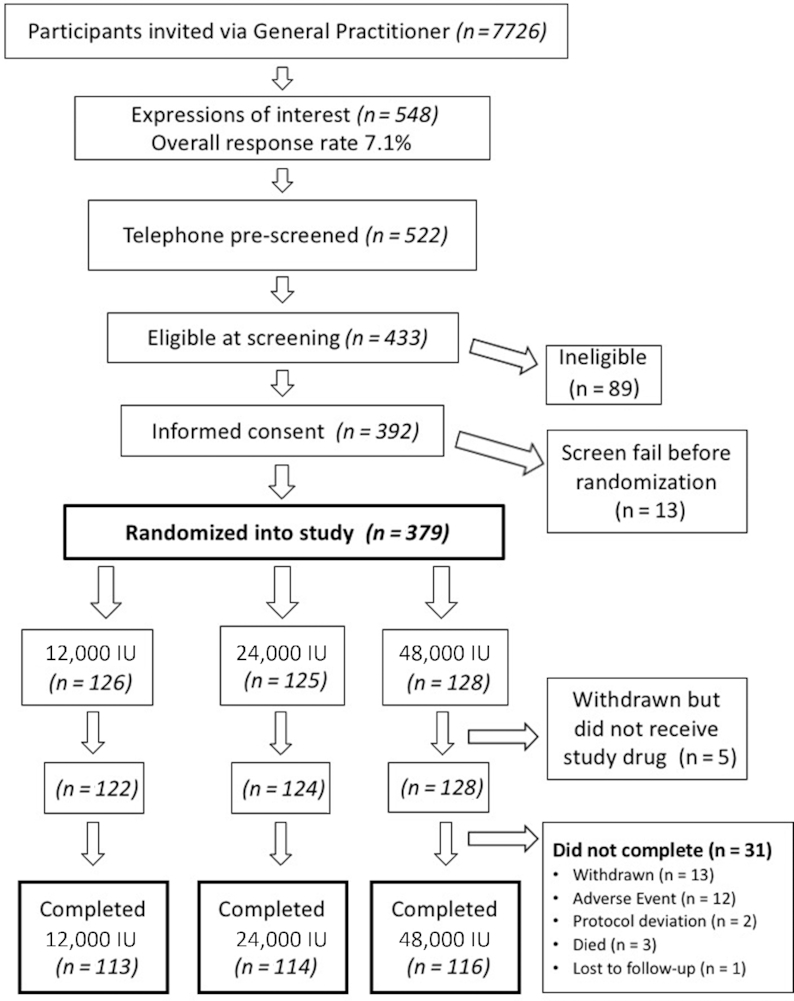
CONSORT diagram showing the progression of participants through the study from invitation by general practitioners to completion. Treatment allocation and number of participants are given. CONSORT, Consolidated Standards of Reporting Trials.

A total of 379 participants were recruited, with 343 (91%) participants completing the study at 12 mo [see **Figure 1**; Consolidated Standards of Reporting Trials (CONSORT) diagram]. Baseline characteristics, including anthropometric parameters, markers of calcium and vitamin D metabolism and intakes, renal function, falls, and major osteoporotic or hip fracture risk, were well balanced across the 3 arms (see **Table 1**). There was also no difference in self-reported habitual sun exposure between the 3 arms at baseline. There were no differences between arms in subsequent dropout from the study. The mean ± SD baseline 25(OH)D concentration was 40.0 ± 20.1 nmol/L.

**TABLE 1:** Baseline characteristics of participants^1^

	12,000 IU				24,000 IU				48,000 IU			
	*n*	Mean	SD	*n* (%)	*n*	Mean	SD	*n* (%)	*n*	Mean	SD	*n* (%)
Age, y	122	74.6	3.9	—	124	75.0	4.3	—	126	75.4	4.4	—
Age category												
≥70 to <71.5 y	—	—	—	32 (26.2)	—	—	—	31 (25.0)	—	—	—	33 (26.2)
≥71.5 to <74 y	—	—	—	34 (27.9)	—	—	—	30 (24.2)	—	—	—	28 (22.2)
≥74 to <77 y	—	—	—	29 (23.8)	—	—	—	32 (25.8)	—	—	—	28 (22.2)
≥77 y	—	—	—	27 (22.1)	—	—	—	31 (25.0)	—	—	—	37 (29.4)
Male gender	126	—	—	69 (54.8)	125	—	—	65 (52.0)	128	—	—	63 (49.2)
Weight, kg	126	73.9	11.8	—	125	77.1	14.5	—	128	76.1	14.2	—
Height, cm	126	167.5	8.1	—	125	167.0	9.9	—	128	167.4	10.0	—
Waist, cm	125	94.5	11.4	—	125	97.7	14.0	—	127	97.5	14.3	—
BMI, kg/m^2^	126	26.3	3.6	—	125	27.5	4.1	—	128	27.1	4.0	—
Fat mass, %	124	23.8	8.5	—	125	25.5	8.2	—	127	24.9	8.3	—
Fat mass trunk, %	124	31.9	8.6	—	125	32.9	7.7	—	127	32.5	7.8	—
Lean mass:fat-free mass, ratio	124	50.0	8.9	—	125	51.6	10.7	—	127	50.7	10.9	—
Bone mineral density												
Total femur. g/cm^2^	120	0.980	0.155	—	118	0.987	0.179	—	125	0.973	0.187	—
Femoral neck, g/cm^2^	121	0.898	0.138	—	122	0.915	0.154	—	123	0.892	0.163	—
Bone area, hip, cm^2^	121	35.6	3.4	—	120	36.1	4.3	—	126	36.2	4.0	—
Serum creatinine, µmol/L	126	81.3	16.3	—	124	81.1	19.8	—	127	83.8	20.8	—
Estimated glomerular filtration rate , mL · min^−1^ · 1.73 m^−2^	122	77.2	14.0	—	123	78.1	16.9	—	125	75.1	17.0	—
Serum urea, mmol/L	124	5.9	1.4	—	122	5.7	1.3	—	121	5.9	1.3	—
Serum calcium, adjusted, mmol/L	126	2.4	0.1	—	125	2.3	0.1	—	127	2.4	0.1	—
Serum 25(OH)D, nmol/L	126	41.6	19.9	—	124	39.5	20.6	—	128	38.9	19.7	—
Free 25(OH)D, pmol/L	126	8.7	4.2	—	124	8.3	4.4	—	127	8.2	4.2	—
Serum 25(OH)D category												
<25 nmol/L	—	—	—	33 (26.2)	—	—	—	34 (27.4)	—	—	—	35 (27.3)
≥25 to <50 nmol/L	—	—	—	52 (41.3)	—	—	—	56 (45.2)	—	—	—	61 (47.7)
≥50 to <75 nmol/L	—	—	—	35 (27.8)	—	—	—	26 (21.0)	—	—	—	24 (18.8)
≥75 nmol/L	—	—	—	6 (4.8)	—	—	—	8 (6.5)	—	—	—	8 (6.3)
Plasma parathyroid hormone, pg/mL	126	48.6	25.7	—	123	47.4	23.3	—	128	50.0	21.3	—
Systolic blood pressure, mm Hg	126	149.5	20.9	—	124	148.0	20.8	—	128	149.2	22.3	—
Diastolic blood pressure, mm Hg	126	74.8	9.7	—	124	75.6	11.2	—	128	74.7	10.0	—
Heart rate, beats/min	126	66.8	12.9	—	124	68.9	11.5	—	128	68.3	11.0	—
Dietary calcium intake, mg/d	119	818.6	358.8	—	121	823.0	398.5	—	123	864.7	406.2	—
Dietary vitamin D, µg/d	119	3.6	2.0	—	121	3.7	2.5	—	123	4.0	3.0	—
No. of falls during previous year^2^												
Median, IQR	118	0	0–0	—	120	0	0–1	—	122	0	0–0	—
Mean, SD	118	0.2	0.6	—	120	0.4	0.8	—	122	0.3	0.6	—
10-y risk,^3^ %												
Major osteoporotic fracture	114	11.3	6.2	—	112	11.6	7.6	—	118	11.8	6.4	—
Hip fracture	114	4.2	3.2	—	112	4.5	4.2	—	118	4.6	3.7	—

^1^Descriptive statistics are presented for the variables from the statistical analysis plan as means and SDs for continuous variables and *n* (%) within each category for categorical variables, except where otherwise stated. No comparative analysis was conducted on baseline values. IU, international units; 25(OH)D, 25-hydroxyvitamin D.

2 Falls are self-reported recall of falls at baseline (whereas a prospective falls diary was used for the duration of the trial; see Table 2 and main text).

3 Ten-year estimated fracture risk (using FRAX) without adjustment for bone mineral density.

There was no significant difference between arms for change in BMD at the total hip site, comparing 12,000 IU with 24,000 IU (*P* = 0.39) or with 48,000 IU (*P* = 0.08). At the femoral neck, there was no significant difference between 12,000 and 24,000 IU (*P* = 0.43) or 48,000 IU (*P* = 0.62) (see **Table 2**). However, after 12 mo, there were significant group differences between arms for change in plasma 25(OH)D (*P* < 0.01 for comparison of the lowest dose with either of the 2 higher doses). At 12 mo, PTH was lower than at baseline for all dosages, with a significant difference in the decrease in PTH over 12 mo between 12,000 and 48,000 IU (*P* < 0.01) but not between 12,000 and 24,000 IU (*P* = 0.78). For details, see Table 2.

**TABLE 2:** BMD, 25(OH)D, and plasma PTH concentrations achieved at 12 mo and changes from baseline^1^

	12,000 IU			24,000 IU			Adjusted ANCOVA comparison: 24,000–12,000 IU,*P*	48,000 IU			Adjusted ANCOVA comparison: 48,000–12,000 IU,*P*
	*n*	Mean	SD	*n*	Mean	SD		*n*	Mean	SD	
BMD hip, g/cm^2^											
12 mo	110	0.977	0.149	108	0.992	0.184		113	0.963	0.165	
Δ	109	−0.001	0.014	106	−0.003	0.018	0.39	112	−0.005	0.016	0.08
BMD FN, g/cm^2^											
12 mo	110	0.897	0.131	110	0.919	0.155		112	0.888	0.146	
Δ	110	0.002	0.020	110	−0.00003	0.024	0.43	111	0.0008	0.021	0.62
25(OH)D, nmol/L											
12 mo	112	55.9	15.6	114	64.6	15.3		113	79.0	15.1	
Δ	112	14.3	12.6	113	25.3	18.1	<0.01	113	40.6	19.9	<0.01
Free 25(OH)D, pmol/L											
12 mo	112	11.7	3.3	113	13.8	3.4		113	16.8	4.3	
Δ	112	3.0	2.7	112	5.5	3.8	<0.01	112	8.7	5.0	<0.01
PTH, pg/mL											
12 mo	112	44.0	21.3	115	44.7	24.4		113	40.2	18.4	
Δ	112	−2.9	18.4	113	−2.9	18.1	0.78	113	−10.6	15.4	<0.01
No. of falls during 12 mo^2^											
Mean, SD	92	0.9	1.9	81	0.7	1.4		88	1.2	2.9	
Median, IQR	92	0	0–1	81	0	0–1		88	0	0–1	0.73^3^

1 Values are means and SDs for achieved values and changes over 12 mo (by arm), except where otherwise stated. BMD, bone mineral density; FN, femoral neck; IU, international units; PTH, parathyroid hormone; 25(OH)D, 25-hydroxyvitamin D; Δ, change from baseline. *P* values for variables at 12 mo. in addition to change were not specified in the analysis plan and so are not presented here; the prespecified outcome was change. The *P* values reported are those for comparing the 24,000 IU (or 48,000 IU) arm with the reference arm of 12,000 IU to test whether any significant difference exists in the change in outcome between baseline and 12 mo when adjusted for covariates as described in the main part of the text.

2 Number of falls reported using a prospective falls diary over 12 mo. Data on 31 participants who did not provide data on the number of falls are not included here. For further details, see the main text.

3 The *P* value for falls represents the chi-square test examining the association between all 3 arms and categorized number of falls (0, 1, 2, 3, ≥4). Fisher's exact test here gives a similar *P* value of 0.7.

With dose of vitamin D included in the model, there were no significant associations of baseline PTH, 25(OH)D, fat mass, height, estimated glomerular filtration rate, or gender with the change in BMD at the total hip site. However, change in total hip BMD was significantly associated with age, which was consistent with a significant decrease, relative to the youngest quartile, in the oldest quartile (aged ≥77 y; *P* = 0.01). Body weight was significantly associated with an increased positive effect on change in BMD at this site (*P* = 0.03) equivalent to ∼0.002 g/cm^2^ per 10-kg difference in body weight. At the femoral neck, baseline BMD (*P* < 0.01), female gender (*P* = 0.03), and higher PTH (*P* = 0.02) were associated with a decrease in BMD. Further exploratory regression modeling demonstrated that a higher C-terminal telopeptide (bCTX) at 12 mo was significantly associated with a decrease in BMD at the total hip (*P* = 0.01), whereas a higher baseline PTH was significantly associated with older age (aged ≥77 y; *P* = 0.02), weight (*P* < 0.01), and lower plasma 25(OH)D (*P* < 0.01). Post hoc analyses found a dose-dependent increase in free 25(OH)D, but baseline free 25(OH)D did not influence ΔBMD in response to the supplement, which was similar to relations seen for total 25(OH)D and ΔBMD. Further post hoc analysis found that baseline plasma 25(OH)D > or <25 nmol/L (as a categorical covariate) did not significantly show an effect of vitamin D supplementation on ΔBMD, nor was there any significant interaction of this term with trial arm.

There was no apparent difference in habitual sun exposure between the doses during the study period. There were no dose effects of supplement on AEs, including fall frequency (see Table 2). A total of 249 falls occurred among the 141 participants who fell. The majority of fallers experienced a first fall in the first 3 mo: 27 of 100 total participants (27%) in the 12,000-IU arm, 19 of 95 (20%) in the 24,000-IU arm, and 27 of 97 (28%) in the 48,000-IU arm. In the second 3 mo, 8%, 13%, and 11% of participants had a first fall; in the third 3 mo, 7%, 5%, and 5%, and in the final 3-mo period, 6%, 7%, and 7% of participants, respectively, fell for the first time. Thus, the proportion who fell in each arm of the study appeared to be similar for all 3-mo blocks. There were no symptomatic cases of renal stones or hypercalcemia, although there were 3 participants who showed an increased serum-adjusted calcium during the study.

**FIGURE 2:**
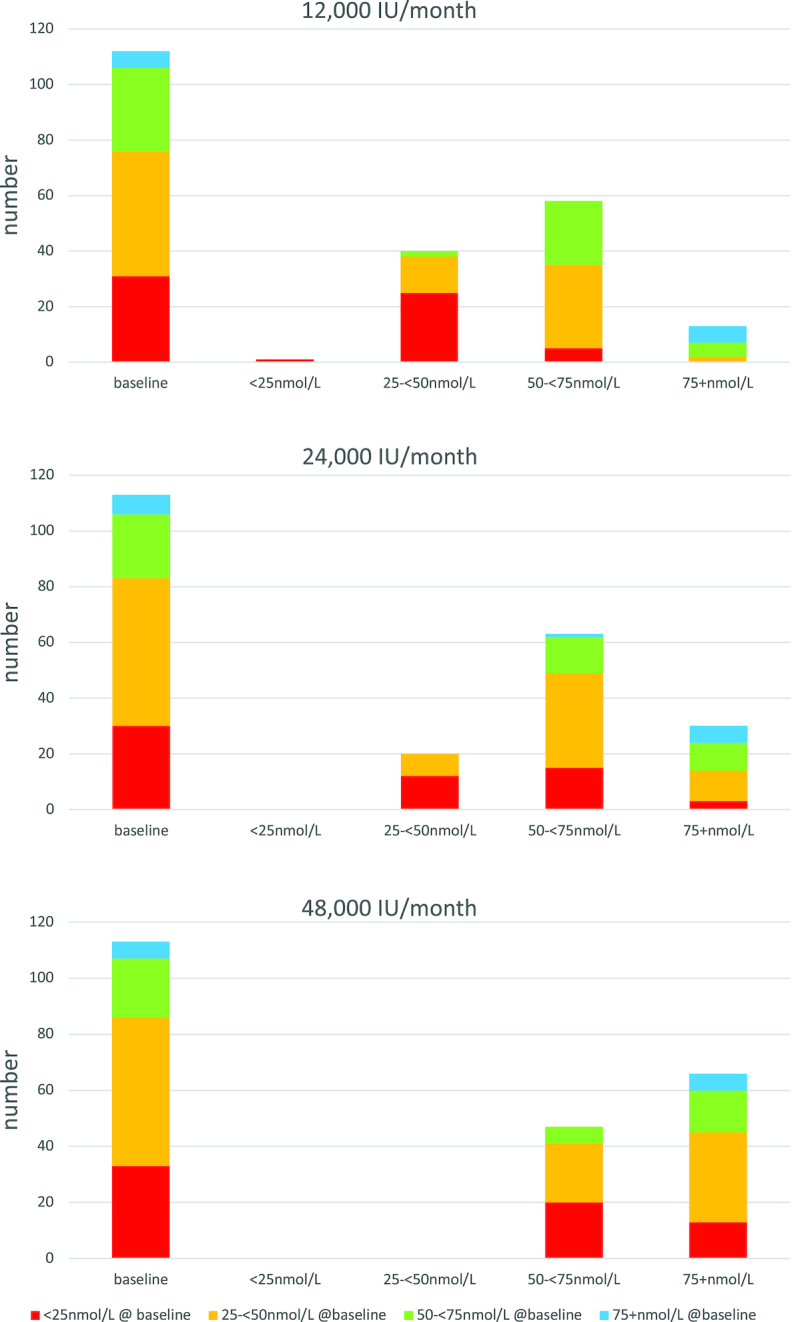
Vitamin D status at baseline is presented by category for each monthly dose of supplement: 12,000, 24,000, and 48,000 IU. The colors reflect the category of vitamin D status at baseline, and the first column shows the number of participants for each category at baseline, with columns 2, 3, 4, and 5 showing the number by category at 12 mo.

The significant increase in mean plasma 25(OH)D with all 3 doses is presented in **Table 3**, and, combining the categories displayed in the table, this demonstrates that 99.7%, 100%, and 100% achieved a level of ≥25 nmol/L at 12 mo with 12,000, 24,000, and 48,000 IU, respectively. The achievement of alternative thresholds of 25(OH)D (including 50 and 75 nmol/L) is also presented in Table 3 and **Figure 2**. The *P* values presented were not adjusted for multiple comparisons. Conclusions around significant *P* values are robust to Bonferroni correction for the change in 25(OH)D, change in free 25(OH)D, and exploratory association between baseline PTH and baseline 25(OH)D results above.

**TABLE 3:** Plasma 25(OH)D concentrations at baseline and 12 mo by dose^1^

		12-mo category, *n* (%)			
Dose and baseline category	Baseline, *n* (%)	<25 nmol/L	25 to <50 nmol/L	50 to <75 nmol/L	≥75 nmol/L
12,000 IU (*n* = 112)					
<25 nmol/L	31 (27.7)	1 (3.2)	25 (80.6)	5 (16.1)	0 (0.0)
25 to <50 nmol/L	45 (40.2)	0 (0)	13 (28.9)	30 (66.7)	2 (4.4)
50 to <75 nmol/L	30 (26.8)	0 (0)	2 (6.7)	23 (76.7)	5 (16.7)
≥75 nmol/L	6 (5.4)	0 (0)	0 (0)	0 (0)	6 (100)
24,000 IU (*n* = 113)					
<25 nmol/L	30 (26.5)	0 (0)	12 (40.0)	15 (50.0)	3 (10.0)
25 to <50 nmol/L	53 (46.9)	0 (0)	8 (15.1)	34 (64.2)	11(20.8)
50 to <75 nmol/L	23 (20.4)	0 (0)	0 (0.0)	13 (56.5)	10 (43.5)
≥75 nmol/L	7 (6.2)	0 (0)	0 (0.0)	1 (14.3)	6 (85.7)
48,000 IU (*n* = 113)					
<25 nmol/L	33 (29.2)	0 (0)	0 (0)	20 (60.6)	13 (39.4)
25 to <50 nmol/L	53 (46.9)	0 (0)	0 (0)	21 (39.6)	32 (60.4)
50 to <75 nmol/L	21 (18.6)	0 (0)	0 (0)	6 (28.6)	15 (71.4)
≥75 nmol/L	6 (5.3)	0 (0)	0 (0)	0 (0.0)	6 (100)

^1^Values are *n* (%) for each category by dose given. For each dose, the last 4 columns present the number (%) ending the study in each category [for participants with 25(OH)D data at both time points]. IU, international units; 25(OH)D, 25-hydroxyvitamin D.

## Discussion

There was no significant dose effect on BMD at the total femur or neck of femur when giving a monthly dose of 12,000, 24,000, or 48,000 IU vitamin D. This is equivalent to a daily dose of 10, 20, or 40 µg (400, 800, and 1600 IU), respectively. The primary objective of this study was to evaluate the dose effect of vitamin D supplementation on the attenuation of the age-associated decline in BMD (12, 19), and our results complement a recent meta-analysis by Reid et al. (1), which concluded that there was no clinically significant effect of vitamin D supplementation on BMD, although that study showed a 0.8% greater BMD at the neck of femur over a mean study duration of 2 y. A more recent study (not included in Reid et al.'s meta-analysis) showed a significant effect of vitamin D supplementation on total hip BMD, at a dose of 25 µg (1000 IU) but not at 10 µg (400 IU)/d, when compared with placebo (19). Of note, the latter study, performed in Aberdeen, United Kingdom, recruited a younger population than ours (mean age: 65 y), comprising only women who also had a lower mean serum 25(OH)D concentration at baseline (34 nmol/L) (19). However, it is difficult to compare the results of either the meta-analysis or the Aberdeen study with the VDOP because both evaluated the effect of vitamin D compared with placebo, whereas the VDOP was a dose-ranging study, with the 10-µg (400-IU)/d equivalent dose as reference.

Our population had concentrations of circulating 25(OH)D comparable to previous UK population surveys, including the National Diet and Nutrition Survey whose participants aged >65 y had an annualized mean plasma 25(OH)D concentration of 43.4 nmol/L in men and 47.9 nmol/L in women, with 21% of men and 9% of women having a plasma 25(OH)D concentration <25 nmol/L. Similar results were also found in the Health Survey for England in 2005 (21, 16). Participants in our study had dietary vitamin D intakes slightly higher than, but comparable to, those seen in the National Diet and Nutrition Survey (2), and overall we believe that our results are generalizable to the United Kingdom.

Our study found an increase in plasma 25(OH)D and free 25(OH)D with supplementation and a decrease in plasma PTH with a dose effect on plasma and free 25(OH)D concentrations. Results for both 25(OH)D and free 25(OH)D were robust to post hoc Bonferroni correction. The lowest dose, equivalent to 400 IU (10 µg)/d, was sufficient to eliminate vitamin D deficiency in 97% of this population, which is consistent with previous studies (22). Meta-regression analyses have found a nonlinear relation between dose of vitamin D and resultant 25(OH)D concentration (3, 22). Autier et al. (23) found that a daily dose of 1 µg vitamin D_3_ resulted in an increase in 25(OH)D of 1.95 nmol/L, although similar doses could result in a 300–400% difference in 25(OH)D concentrations, presumably related to differing characteristics between participants, which may be related to factors that include season (24), gender (25), body weight (26), obesity (25), and dose interval (3), as well as the dose itself, treatment duration, age, and the 25(OH)D concentration at outset (27). In our study, the dose-response was also nonlinear, with an increase in 25(OH)D per microgram of vitamin D_3_/d of 1.43, 1.27, and 1.00 nmol/L for 12,000-, 24,000-, and 48,000-IU doses, respectively, which was less than has been reported elsewhere (3, 22, 23, 27). Possible explanations for this include altered pharmacokinetics of vitamin D after daily compared with monthly dosing, leading to differences in tissue distribution, hydroxylation rate to 25(OH)D, and catabolism. Also, the fact that there was a month between dosing and collection of trough blood samples may have resulted in an apparently lower 25(OH)D concentration compared with daily dosing.

Our study does not show an effect of dose of vitamin D on BMD, but it is possible that all 3 doses attenuated an anticipated decrease in BMD of ∼0.6% over this period (19) because we had no placebo comparator. An alternative explanation for studies showing a positive effect of vitamin D on BMD may be the treatment of a undetected osteomalacia, which would result in an increase in BMD (9). In the VDOP study, although baseline 25(OH)D concentration was 6.2 nmol/L higher than in the study by MacDonald et al. (19), we still found that 28% had a baseline serum 25(OH)D <25 nmol/L, which might indicate a risk of osteomalacia and is a similar proportion to previous UK surveys (21, 16). However, baseline serum 25(OH)D was not a significant predictor of response to treatment, and although others have suggested that an increase in BMD to vitamin D treatment may only be found in those with a low baseline 25(OH)D (28), in post hoc statistical analysis we found that baseline serum 25(OH)D <25 nmol/L was not a significant predictor of response to treatment. However, our study was not powered for this subgroup analysis and studies specifically designed to address this hypothesis are required.

Reporting of AEs was according to International Council for Harmonization of Technical Requirements for Human Use (ICH) guidance. Twelve participants withdrew from the study because of AEs, but only 1 of these withdrawals was related to the intervention (“feeling unwell related to vitamin D”). There were low rates of hypercalcemia and urolithiasis, consistent with the rates expected for this population, with no evidence of a dose effect (29, 30) and no overall difference in the frequency of AEs between the vitamin D doses.

It has been suggested that vitamin D supplementation at doses of 20–25 μg (800–1000 IU)/d decreases the incidence of falls and fractures in older women, whereas lower doses are ineffective (31–33). In contrast, other meta-analyses, including the latest Cochrane Review (34), stress that certain characteristics of both the population studied and the intervention used influence efficacy, with studies in older and frail people living in care homes who were coprescribed calcium with vitamin D supplementation showing a lower risk of hip and other nonvertebral fracture. In our study, approximately half of participants reported a fall over 12 mo, which is slightly greater than the estimated 32–42% reported for this age group (35) but comparable with other vitamin D studies (36). However, no effect of dose of vitamin D on the rate of falls was seen. Focusing on studies in older people, a very high dose of an oral supplement of 12,500 µg (500,000 IU) given annually has been associated with an increased risk of falls (11), whereas a single intramuscular injection of 7500 µg (300,000 IU) vitamin D_2_ given to care-home residents had no effect on hip fracture incidence over 10 mo of follow-up (37). Another study giving an annual oral dose of 7500 µg (300,000 IU) vitamin D_2_ for 3 y found an increase in hip fracture risk (38), although Trivedi et al. (39) found that 100,000 IU vitamin D_3_ administered orally every 4 mo was associated with a lower fracture rate. A recent study found that a monthly oral dose of 1500 µg (60,000 IU) vitamin D_3_ was associated with almost 20% more falls than 600 µg (24,000 IU)/mo (40), whereas another study in a younger population, using a loading dose of 5000 µg (200,000 IU) followed by 2500 µg (100,000 IU)/mo, did not show any effect on falls or fracture rate during an average of 3.5 years of follow-up (41). Finally, a study using a range of daily doses of vitamin D_3_, from placebo to 120 µg (4800 IU) in postmenopausal women showed a significantly lower falls rate in participants taking 40–80 µg (1600–3200 IU) vitamin D_3_/d (*P* = 0.048) than the highest or lowest doses (36). Thus, the evidence from clinical trials remains conflicting, and other factors such as the falls rate before recruitment and baseline 25(OH)D concentration may be of significance.

We conclude that there was no difference in ΔBMD between the 3 dosages of vitamin D, which suggests no effect of the intervention or that all doses may have attenuated the anticipated decrease in BMD over 12 mo. Monthly dosing with oral vitamin D_3_ is a safe and effective strategy to increase plasma 25(OH)D >25 nmol/L. This is achieved with all 3 doses in the majority of people and aligns with the current recommendation by the SACN. The lack of a dose effect on adverse outcomes is reassuring.

## Notes

Supported by Arthritis Research UK (Clinical Studies grant 19544) and also supported by the Medical Research Council, UK (MRC program no. U105960371). The study design was internationally peer-reviewed on behalf of Arthritis Research UK as part of the funding-decision process. Reviewers’ recommendations were taken into consideration in the design of the trial. Arthritis Research UK had no involvement in the analyses or publication of the results of the trial. Newcastle upon Tyne Hospitals Trust was the sponsor for the study.

Abbreviations used: AE, adverse event; BMD, bone mineral density; IOM, Institute of Medicine; IU, international units;PTH, parathyroid hormone; RDA, Recommended Dietary Allowance; SACN, Scientific Advisory Committee on Nutrition; VDOP, Vitamin D Supplementation in Older People; 25(OH)D, 25-hydroxyvitamin D; 25(OH)D_2_, 25-hydroxyvitamin D_2_; 25(OH)D_3_, 25-hydroxyvitamin D_3_.
